# An Address to the Graduating Class in the University of Nashville

**Published:** 1855-05

**Authors:** 


*An Address to the Graduating Class in the University of Nashville.* By PAUL F. EVE, M. D.

"Public opinion being unenlightened in medicine, physicians should not be influenced by it."

This is the text of Prof. Eve's discourse, and, as might be expected from it, it is a vigorous assertion of the right of the medical mind to govern medical ethics and opinions. We ought to thank Prof. Eve for the compliment he pays us, in quoting from a forgotten article of our own, a lashing of a meddlesome clergyman, which we now recollect to have administered with great unction, and comfort to our inner man. H.

